# Premature Mortality Excess Rates Before and During the COVID-19 Pandemic: A Comparative Analysis Conducted in Bihor County, Romania

**DOI:** 10.7759/cureus.60403

**Published:** 2024-05-16

**Authors:** Diana Rahota, Razvan G Rahota, Andreea Camarasan, Mihaela M Muresan, Sorina Magheru, Daniela Rahota, Gineta Andreescu, Florin Maghiar, Ovidiu Pop

**Affiliations:** 1 Department of Morphological Disciplines, Faculty of Medicine and Pharmacy, University of Oradea, Oradea, ROU; 2 Department of Medical Disciplines, Faculty of Medicine and Pharmacy, University of Oradea, Oradea, ROU

**Keywords:** bihor county, gross rate, covid-19, excess premature mortality, potential years of life lost

## Abstract

Background: Estimating the excess of premature deaths (before the age of 75 years) and Potential Years of Life Lost allows ranking causes of death as an expression of the burden of disease in a population. We statistically analysed the impact of the coronavirus disease 2019 (COVID-19) pandemic on excess premature mortality in the total population and specifically, by sexes, compared to the pre-pandemic period, through Potential Years of Life Lost.

Material and method: In this retrospective descriptive observational study, we counted excess of premature mortality in the years 2020, 2021, and 2022 by cause of death (cardiovascular diseases, cancer, digestive diseases, injury, COVID-19, and other causes) and by sexes compared to the period average from 2017-2019, based on the deaths registered in Bihor County (48,948 people).

Results: Premature deaths due to COVID-19 (1,745 people of both sexes) contributed 71.3% to excess mortality, the population being similar for both sexes (71.4% in men and 71.2% in women). The Potential Years of Life Lost/death due to COVID-19 was 11.84 years for both sexes (11.76 years in men and 12.02 years in women). Potential Years of Life Lost/all-cause heath was lower during the pandemic (13.42 years for both sexes, 14.06 years for men and 12.32 years for women) compared to the pre-pandemic period (14.6 years for both sexes, 15.1 years for men and 13.5 years for women).

Conclusions: The excess of premature mortality and decreased Potential Years of Life Lost/death during the pandemic, shows an increase in the proportion of deaths at ages closer to the established limit for premature mortality (75 years) compared to the pre-pandemic period.

## Introduction

Recent WHO (World Health Organisation) data on COVID-19 showed over 774,000,000 confirmed cases and more than 7,000,000 deaths worldwide [1]. In Europe, this condition led to death in over 2,250,000 cases. The highest number of new deaths in March 2024 was reported by Russia, followed by the United Kingdom and Sweden [1]. In Romania, the last data showed over 68,000 deaths due to COVID-19 from the beginning of the pandemic until the end of December 2022, with the total number of deaths being above 67,000. When COVID-19 infection coexists with pre-existing comorbidities such as hypertension, cardiovascular disease, diabetes, chronic kidney disease, malignancy, or immune-related disorders, the associated mortality rate tends to be higher [2-4].

Potential Years of Life Lost (PYLL) is a measurement tool for premature mortality. PYLL considers both the number of deaths and the age at which the deaths occur, and it takes into account an individual, a group of people, or a population who died prematurely, prior to a selected age limit [5]. Potential Life Years Lost are considered an indicator that can be used in health planning and that allows for the causes of death to be ranked [5]. PYLL effectiveness is attested by the latest studies which show premature mortality [6], related to COVID-19, making it the only tool that can be used for understanding the burden of this virus.

From the beginning of the pandemic until 31.12.2022, on Romania’s territory, 3,312,085 cases of infection with SARS-CoV-2 were registered [7]. In the same time interval, according to existing data on Bihor Country, 92,483 cases of infection with SARS-CoV-2 were diagnosed, the infection with COVID-19 being the main cause of death in 2,919 cases [8].

Given the existing studies in specialized literature demonstrating that COVID-19 infection, along with various comorbid conditions, correlates with higher mortality rates [2-4], we considered it essential to conduct this study to investigate whether these comorbidities also influence the potential differences in the PYLL between pre-pandemic and pandemic periods. Therefore, the present study aims to identify potential differences in PYLL (total, sexes, excess) between the pre-pandemic period (2017-2019) and the pandemic period (2020-2022) using the database from Bihor County (Romania).

## Materials and methods

The study conducted is a retrospective, descriptive, observational epidemiological one. Data were collected from The National Statistical Database, “The Statistical Death Form”, with anonymized data, being approved by The Research Ethical Committee Decision, number CEFMF/03/31.01.2021. The database includes all deaths that occurred in a time interval between January 2017 and December 2022, registered in Bihor County, Romania. For each case, we gathered information on date of birth, date of death, age, sex, address (place of residence), place of death, causes of death, main cause of death, and secondary causes of death. The inclusion criterion was set up by the age limit, 75 years old being the upper limit, which was established after thorough searches, according to more public organizations (WHO, Organization for Economic Co-operation and Development (OECD) and the European Commission); deaths situated below this age were considered premature deaths; no lower limit was established. For fairness and equality, we set the age limit for which a death is considered premature at the age of 75. According to the OECD and the European Commission, life expectancy at birth in Romania in 2019 was 75.6 years, and in 2020, it decreased to 74.2 years [9]. According to WHO/EURO, life expectancy at birth in Romania is 75.4 years (both sexes), 79.1 years for women and 71.8 years for men [10].

The literature cites several ways in which PYLL can be obtained. One possible way to obtain PYLL is based on life table norms for years of life lost, which is the number of years lost by an “average” person in a given population [11]. Another way for obtaining PYLL is by calculating the difference between the potential life limit and the age at death of an individual, which results in the PYLL if the individual dies before the potential life limit [12]. The sum of potential life years lost by all individuals in a community represents the total PYLL in that community (country, county, locality). When we take into consideration the calculation of PYLL, adjustments can be applied by excluding from the calculation, the cases with certain associated comorbidities [11,13] or even deaths that occurred before the age of 1 year. To ensure data comparability, “transparency in documenting the exact method used to calculate years of life lost (YLL)” [13] should be ensured despite all existing and identifiable limitations.

Statistical analysis was performed using Microsoft Excel. From each year’s mortality database, deaths under 75 years of age were extracted in a separate spreadsheet. Two additional columns have been introduced in the spreadsheet with deaths occurring at age <75 years: cause groups according to the International Statistical Classification of Diseases and Related Health Problems (ICD)-10) [14] and potential life years lost per death. Starting from ICD-10, six groups of causes of death were used in the study: Diseases of the circulatory system (I00-I99), Cancer (C00-C-48), Diseases of the digestive system (K00-K93), Injuries (S00-T98), COVID-19 (U71-U72), other (all codes not included in other groups).

The calculation of PYLL performed by our study involved the following formula: *PYLL = 75 - Age at death (in years)*

The sum of PYLL values for all deaths provided the annual PYLL. In order to calculate the overall PYLL and classify it according to sexes and specific causes of death groups, while also determining the total number of deaths and the average age within each cause of death group, the PivotTable function was utilized for each studied year.

The Gross Rate of PYLL (annual) was obtained by using the formula:* (PYLL/Population under 75 years old)X 100.000*

For age groups under 75 years, for each year studied, data was provided from The National Institute of Statistics in Romania [15,16]. The main result of the study was to establish the excess PYLL during the pandemic period (2020-2022) compared to the pre-pandemic reference period (2017-2019). Subtraction between PYLL by cause and sex in each pandemic year and PYLL in the reference period represented excess PYLL.

## Results

**Figure 1:**
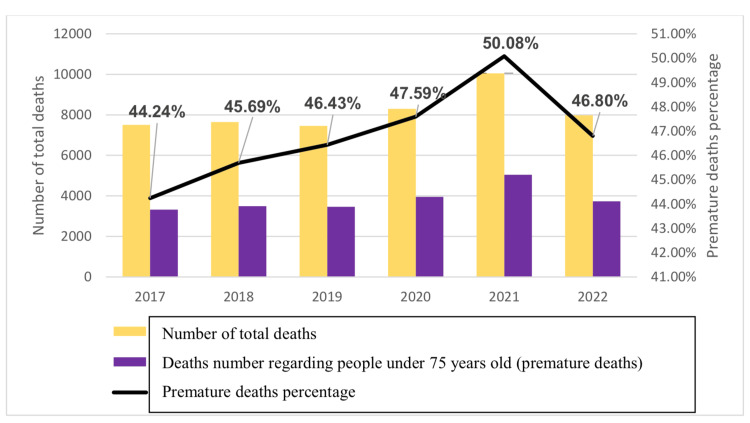
Evolution of premature deaths during pre-COVID-19 pandemic and COVID-19 pandemic periods COVID-19: Coronavirus disease 2019

In our study 48,948 people were included: 25,242 men and 23,700 women, with a sex ratio of 106.50 (men/women=1.065). Figure 1 displays the percentage of premature deaths related to the total deaths for each year examined in our study. We observed a higher incidence of premature deaths during the pandemic years, the highest value being recorded in 2021 followed by 2020 and 2022, compared to the pre-pandemic period (Figure 1).

**Figure 2:**
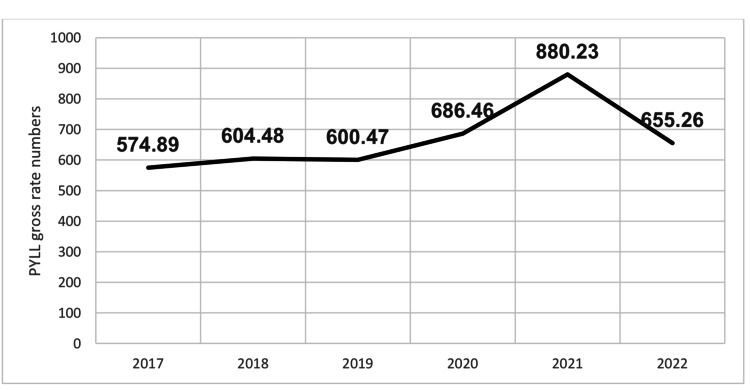
Gross rate of PYLL in the studied population PYLL: Potential Years of Life Lost

Figure 2 illustrates the gross rate of PYLL. The peak of the PYLL gross rate during the pandemic years occurred in 2021 (880), whereas the highest value in the pre-pandemic period was recorded in 2018 (604). A substantial difference between the COVID-19 pandemic and pre-pandemic periods was observed, PYLL gross rate being higher in 2020, 2021, and 2022 compared with 2017, 2018, and 2019 (Figure 2).

The total and sexes-specific number of deaths and PYLL by cause of death in the studied population are presented in Table 1.

**Table 1:** Excess of premature deaths and PYLL, total and by cause, compared with premature deaths and PYLL from 2017-2019, total and by gender PYLL: Potential Years of Life Lost, NA: Data not available, n: number, %: percentage, CVD: Cardiovascular disease

Causes of deaths both sexes/men/women	Average baseline 2017-2019					Excess 2020-2022			
	Premature deaths		PYLL		PYLL/ death	Premature deaths		PYLL	
Both sexes	n	%	n	%		n	%	n	%
All deaths	3425	100%	49889	100%	14.6	2448	71.5%	22923	41.4%
CVD	1381	40.3%	15591	31.3%	11.3	814	58.9%	6402	41.1%
Cancer	1014	29.6%	18782	37.6%	18.5	-345	-34.0%	-22604	-120.3%
Digestive	280	8.2%	3257	6.5%	11.6	13	4.6%	2992	91.9%
Injuries	223	6.5%	3829	7.7%	17.2	-59	-26.5%	3773	98.5%
Other	527	15.4%	8430	16.9%	16.0	280	53.1%	9187	109.0%
COVID-19	NA	NA	NA	NA	NA	1745	NA	20921	NA
Deaths excluding COVID-19	3425	100%	49889	100%	14.6	703	20.5%	-250	-0.5%
Men									
All deaths	2239	100%	33897	100%	15.1	1338	59.8%	13670	40.3%
CVD	902	40.3%	10743	31.7%	11.9	537	59.5%	5571	51.9%
Cancer	649	29.0%	12260	36.2%	18.9	-341	-52.5%	-17262	-140.8%
Digestive	180	8.0%	2297	6.8%	12.8	74	41.1%	2731	118.9%
Injuries	164	7.3%	3105	9.2%	18.9	12	7.3%	3487	112.3%
Other	344	15.4%	5492	16.2%	16.0	101	29.4%	5208	94.8%
COVID-19	NA	NA	NA	NA	NA	955	NA	13935	NA
Deaths excluding COVID-19	2239	100%	33897	100.0%	15.1	383	17.1%	-265	-0.8%
Women									
All deaths	1186	100%	15992	100%	13.5	1110	93.6%	9253	57.9%
CVD	479	40.4%	4848	30.3%	10.1	277	57.8%	831	17.1%
Cancer	365	30.8%	6522	40.8%	17.9	-4	-1.1%	-5342	-81.9%
Digestive	100	8.4%	959	6.0%	9.6	-61	-61%	264	27.5%
Injuries	59	5.0%	725	4.5%	12.3	-71	-120.3%	283	39%
Other	183	15.4%	2938	18.4%	16.1	179	97.8%	3979	135.4%
COVID-19	NA	NA	NA	NA	NA	790	NA	9238	NA
Deaths excluding COVID-19	1186	100.0%	15992	100,0%	13.5	320	27%	15	0.1%

Premature mortality data leading to mean baseline for the years 2017-2019 are presented in Table 2. The results obtained when calculating the PYLL indicators and the difference from the average baseline are presented in Table 3 (for the year 2020),Table 4 (for the year 2021) and Table 5 (for the year 2022).

**Table 2:** Premature mortality indicators 2017-2019, by years and mean baseline, in the studied population PYLL: Potential Years of Life Lost, NA: Data not available, CVD: Cardiovascular disease, COVID-19: Coronavirus disease 2019

Causes of death in both sexes/men/women	2017			2018			2019			Average baseline 2017-2019		
	Premature deaths	PYLL	PYLL/Death	Premature deaths	PYLL	PYLL/ Death	Premature deaths	PYLL	PYLL/ Death	Premature deaths	PYLL	PYLL/ Death
Both sexes												
All deaths	3322	48197	14.51	3491	50838	14.56	3462	50631	14.62	3425	49889	14.57
CVD	1379	15222	11.04	1365	15454	11.32	1398	16096	11.51	1381	15591	11.29
Cancer	987	12846	13.02	1088	14422	13.26	966	29078	30.10	1014	18782	18.52
Digestive	291	4340	14.91	273	4212	15.43	276	1218	4.41	280	3257	11.63
Injuries	211	5661	26.83	223	5519	24.75	236	308	1,.1	223	3829	17.17
Other	454	10128	22.31	542	11231	20.72	586	3931	6.71	527	8430	16.00
COVID-19	NA	NA	NA	NA	NA	NA	NA	NA	NA	NA	NA	NA
Deaths excluding COVID-19	3322	48197	14.51	3491	50838	14.56	3462	50631	14.62	3425	49889	14.57
Men												
All deaths	2152	32356	15.04	2302	34915	15.17	2263	34420	15.21	2239	33897	15.14
CVD	895	10455	11.68	875	10927	12.49	936	10847	11.59	902	10743	11.91
Cancer	590	7551	12.80	672	8799	13.09	685	20429	29.82	649	12260	18.89
Digestive	192	3133	16.32	194	3089	15.92	153	670	4.38	180	2297	12.76
Injuries	168	4455	26.52	192	4694	24.45	132	165	1.25	164	3105	18.93
Other	307	6762	22.03	369	7406	20.07	357	2309	6.47	344	5492	15.97
COVID-19	NA	NA	NA	NA	NA	NA	NA	NA	NA	NA	NA	NA
Deaths excluding COVID-19	2152	32356	15.04	2302	34915	15.17	2263	34420	15.21	2239	33897	15.14
Women												
All deaths	1170	15841	13.54	1189	15923	13.39	1199	16211	13.52	1186	15992	13.48
CVD	484	4767	9.85	490	4527	9.24	462	5249	11.36	479	4848	10.12
Cancer	397	5295	13.34	416	5623	13.52	281	8649	30.78	365	6522	17.87
Digestive	99	1207	12.19	79	1123	14.22	123	548	4.46	100	959	9.59
Injuries	43	1206	28.05	31	825	26.61	104	143	1.38	59	725	12.29
Other	147	3366	22.90	173	3825	22.11	229	1622	7.08	183	2938	16.05
COVID-19	NA	NA	NA	NA	NA	NA	NA	NA	NA	NA	NA	NA
Deaths excluding COVID-19	1170	15841	13.54	1189	15923	13.39	1199	16211	13.52	1186	15992	13.48

**Table 3:** Indicators of premature mortality in 2020 and the difference (excess) from the mean baseline in the studied population PYLL: Potential Years of Life Lost, NA: Data not available, n: number, %: percentage, CVD: Cardiovascular disease, COVID-19: Coronavirus disease 2019

Causes of death in both sexes/men/women	Average baseline 2017-2019					2020					Difference compared with an average baseline			
	Premature deaths		PYLL		PYLL/ death	Premature deaths		PYLL		PYLL/ death	Premature deaths		PYLL	
	n	%	n	%		n	%	n	%		n	%	n	%
Both sexes														
All deaths	3425	100%	49889	100%	14.6	3948	100%	53612	100%	13.6	523	15.3%	3723	7.5%
CVD	1381	40.3%	15591	31.3%	11.3	1570	39.8%	17042	31.8%	10.9	189	13.7%	1451	9.3%
Cancer	1014	29.6%	18782	37.6%	18.5	927	23.5%	11734	21.9%	12.7	-87	-8.6%	-7048	-37.5%
Digestive	280	8.2%	3257	6.5%	11.6	259	6.6%	3735	7.0%	14.4	-21	-7.5%	478	14.7%
Injuries	223	6.5%	3829	7.7%	17.2	176	4.5%	4340	8.1%	24.7	-47	-21.1%	511	13.3%
Other	527	15.4%	8430	16.9%	16.0	578	14.6%	11059	20.6%	19.1	51	9.7%	2629	31.2%
COVID-19	NA	NA	NA	NA	NA	438	11.1%	5702	10.6%	13.0	438	NA	5702	NA
Deaths excluding COVID-19	3425	100%	49889	100%	14.6	3510	88.9%	47910	89.4%	13.6	85	2.5%	-1979	-4.0%
Men														
All deaths	2239	100%	33897	100%	15.1	2550	100%	36155	100%	14.2	311	13.9%	2258	6.7%
CVD	902	40.3%	10743	31.7%	11.9	1036	40.6%	12153	33.6%	11.7	134	14.9%	1410	13.1%
Cancer	649	29.0%	12260	36.2%	18.9	556	21.8%	7226	20.0%	13.0	-93	-14.3%	-5034	-41.1%
Digestive	180	8.0%	2297	6.8%	12.8	188	7.4%	2752	7.6%	14.6	8	4.4%	455	19.8%
Injuries	164	7.3%	3105	9.2%	18.9	153	6.0%	3739	10.3%	24.4	-11	-6.7%	634	20.4%
Other	344	15.4%	5492	16.2%	16.0	348	13.6%	6835	18.9%	19.6	4	1.2%	1343	24.5%
COVID-19	NA	NA	NA	NA	NA	269	10.5%	3450	9.5%	12.8	269	NA	5702	NA
Deaths excluding COVID-19	2239	100%	33897	100%	15.1	2281	89.5%	32705	90.5%	14.3	42	1.9%	-1192	-3.5%
Women														
All deaths	1186	100%	15992	100,0%	13.5	1398	100%	17457	100%	12.5	212	17.9%	1465	9.2%
CVD	479	40.4%	4848	30,3%	10.1	534	38.20%	4889	28.01%	9.2	55	11.5%	41	0.8%
Cancer	365	30.8%	6522	40,8%	17.9	371	26.54%	4508	25.82%	12.2	6	1.6%	-2014	-30.9%
Digestive	100	8.4%	959	6,0%	9.6	71	5.08%	983	5.63%	13.8	-29	-29.0%	24	2.5%
Injuries	59	5.0%	725	4,5%	12.3	23	1.65%	601	3.44%	261	-36	-61.0%	-124	-17.1%
Other	183	15.4%	2938	18,4%	16.1	230	16.45%	4224	24.20%	18.4	47	25.7%	1286	43.8%
COVID-19	NA	NA	NA	NA	NA	169	12.09%	2252	12.90%	13.3	169	NA	2252	NA
Deaths excluding COVID-19	1186	100%	15992	100,0%	13.5	1229	87.91%	15205	87.10%	12.4	43	3.6%	-787	-4.9%

**Table 4:** Indicators of premature mortality in the year 2021 and the difference (excess) from the mean baseline in the studied population PYLL: Potential Years of Life Lost, NA: Data not available, n: number, %: percentage, CVD: Cardiovascular disease, COVID-19: Coronavirus disease 2019

Causes of deaths both sexes/men/women	Average baseline 2017-2019					2021					Difference compared with average baseline			
	Premature deaths		PYLL		PYLL/ death	Premature deaths		PYLL		PYLL/ death	Premature deaths		PYLL	
	n	%	n	%		n	%	n	%		n	%	n	%
Both sexes														
All deaths	3425	100%	49889	100%	14.6	5042	100%	65903	100%	13.1	1617	47.2%	16014	32.1%
CVD	1381	40.3%	15591	31.3%	11.3	1868	37.0%	19648	29.8%	10.5	487	35.3%	4057	26.0%
Cancer	1014	29.6%	18782	37.6%	18.5	890	17.7%	11040	16.8%	12.4	-124	-12.2%	-7742	-41.2%
Digestive	280	8.2%	3257	6.5%	11.6	277	5.5%	4177	6.3%	15.1	-3	-1.1%	920	28.2%
Injuries	223	6.5%	3829	7.7%	17.2	222	4.4%	5910	9.0%	26.6	-1	-0.4%	2081	54.3%
Other	527	15.4%	8430	16.9%	16.0	671	13.3%	11971	18.2%	17.8	144	27.3%	3541	42.0%
COVID-19	NA	NA	NA	NA	NA	1114	22.1%	13157	20.0%	11.8	1114	NA	13157	NA
Deaths excluding COVID-19	3425	100%	49889	100%	14.6	3928	77.9%	52746	80.0%	13.4	503	14.7%	2857	5.7%
Men														
All deaths	2239	100%	33897	100%	15.1	3096	100%	42875	100%	13.8	857	38.3%	8978	26.5%
CVD	902	40.3%	10743	31.7%	11.9	1199	38.7%	13739	32.0%	11.5	297	32.9%	2996	27.9%
Cancer	649	29.0%	12260	36.2%	18.9	532	17.2%	6292	14.7%	11.8	-117	-18.0%	-5968	-48.7%
Digestive	180	8.0%	2297	6.8%	12.8	197	6.4%	3115	7.3%	15.8	17	9.4%	818	35.6%
Injuries	164	7.3%	3105	9.2%	18.9	187	6.0%	5113	11.9%	27.3	23	14.0%	2008	64.7%
Other	344	15.4%	5492	16.2%	16.0	407	13.1%	7511	17.5%	18.5	63	18.3%	2019	36.8%
COVID-19	NA	NA	NA	NA	NA	574	18.5%	7105	16.6%	12.4	574	NA	7105	NA
Deaths excluding COVID-19	2239	100%	33897	100%	15.1	2522	81.5%	35770	83.4%	14.2	283	12.6%	1873	5.5%
Women														
All deaths	1186	100%	15992	100%	13.5	1946	100%	23028	100%	1.8	760	64.1%	7036	44.0%
CVD	479	40.4%	4848	30.3%	10.1	669	34.4%	5909	25.7%	8.8	190	39.7%	1061	21.9%
Cancer	365	30.8%	6522	40.8%	17.9	358	18.4%	4748	20.6%	13.3	-7	-1.9%	-1774	-27.2%
Digestive	100	8.4%	959	6.0%	9.6	80	4.1%	1062	4.6%	13.3	-20	-20.0%	103	10.7%
Injuries	59	5.0%	725	4.5%	12.3	35	1.8%	797	3.5%	22.8	-24	-40,7%	72	9.9%
Other	183	15.4%	2938	18.4%	16.1	264	13.6%	4460	19.4%	16.9	81	44,3%	1522	51.8%
COVID-19	NA	NA	NA	NA	NA	540	27.7%	6052	26.3%	11.2	540	NA	6052	NA
Deaths excluding COVID-19	1186	100%	15992	100%	13.5	1406	72.3%	16976	73.7%	12.1	220	18.5%	984	6.2%

**Table 5:** Indicators of premature mortality in 2022 and the difference (excess) from the mean baseline in the studied population PYLL: Potential Years of Life Lost, NA: Data not available, n: number, %: percentage, CVD: Cardiovascular disease, COVID-19: Coronavirus disease 2019

Causes of death in both sexes/men/women	Average baseline 2017-2019					2022					Difference compared with an average baseline			
	Premature deaths		PYLL		PYLL/ death	Premature deaths		PYLL		PYLL/ death	Premature deaths		PYLL	
	n	%	n	%		n	%	n	%		n	%	n	%
Both sexes														
All deaths	3425	100%	49889	100%	14.6	3733	100%	50823	100%	13.6	308	9.0%	934	1.9%
CVD	1381	40.3%	15591	31.3%	11.3	1519	40.7%	16485	32.4%	10.9	138	10.0%	894	5.7%
Cancer	1014	29.6%	18782	37.6%	18.5	880	23.6%	10968	21.6%	12.5	-134	-13.2%	-7814	-41.6%
Digestive	280	8.2%	3257	6.5%	11,6	317	8.5%	4851	9.5%	15.3	37	13.2%	1594	48.9%
Injuries	223	6.5%	3829	7.7%	17.2	212	5.7%	5010	9.9%	23.6	-11	-4.9%	1181	30.8%
Other	527	15.4%	8430	16.9%	16.0	612	16.4%	11447	22.5%	18.7	85	16.1%	3017	35.8%
COVID-19	NA	NA	NA	NA	NA	193	5.2%	2062	4.1%	10.7	193	NA	2062	NA
Deaths excluding COVID-19	3425	100%	49889	100%	14.6	3540	94.8%	48761	95.9%	13.8	115	3.4%	-1128	-2.3%
Men											0			
All deaths	2239	100%	33897	100%	15.1	2409	100%	34079	100%	14.1	170	7.6%	182	0.5%
CVD	902	40.3%	10743	31.7%	11.9	1008	41.8%	11908	34.9%	11.8	106	11.8%	1165	10.8%
Cancer	649	29.0%	12260	36.2%	18.9	518	21.5%	6000	17.6%	11.6	-131	-20.2%	-6260	-51.1%
Digestive	180	8.0%	2297	6.8%	12.8	229	9.5%	3755	11.0%	16.4	49	27.2%	1458	63.5%
Injuries	164	7.3%	3105	9.2%	18.9	164	6.8%	3950	11.6%	24.1	0	0.0%	845	27.2%
Other	344	15.4%	5492	16.2%	16.0	378	15.7%	7338	21.5%	19.4	34	9.9%	1846	33.6%
COVID-19	NA	NA	NA	NA	NA	112	4.6%	1128	3.3%	10.1	112	NA	1128	NA
Deaths excluding COVID-19	2239	100%	33897	100%	15.1	2297	95.4%	32951	96.7%	14.3	58	2.6%	-946	-2.8%
Women														
All deaths	1186	100%	15992	100%	13.5	1324	100%	16744	100%	12.6	138	11.6%	752	4.7%
CVD	479	40.4%	4848	30.3%	10.1	511	38.6%	4577	27.3%	9.0	32	6.7%	-271	-5.6%
Cancer	365	30.8%	6522	40.8%	17.9	362	27.3%	4968	29.7%	13.7	-3	-0.8%	-1554	-23.8%
Digestive	100	8.4%	959	6.0%	9.6	88	6.6%	1096	6.5%	12.5	-12	-12.0%	137	14.3%
Injuries	59	5.0%	725	4.5%	12.3	48	3.6%	1060	6.3%	22.1	-11	-18.6%	335	46.2%
Other	183	15.4%	2938	18.4%	16.1	234	17.7%	4109	24.5%	17.6	51	27.9%	1171	399%
COVID-19	NA	NA	NA	NA	NA	81	6.1%	934	5.6%	11.5	81	NA	934	NA
Deaths excluding COVID-19	1186	100.0%	15992	100%	13.5	1243	93.9%	15810	94.4%	12.7	57	4.8%	-182	-1.1%

The excess of premature deaths in the period 2020-2022 in the population of Bihor Country (Table 1) was 2,448 deaths (of which 54.66% in men and 45.34% in women) which led to a total of 22,923 potential years of life loss (of which 59.63% in men and 40.37% in women). When analysing the excess by causes of death in both sexes compared to the reference period, a decrease in the number of deaths from cancer (-34%) and injuries (-26.5%) was found, while the PYLL for these two causes registered a decrease for cancer (-120.3%) and an increase for injuries (98.5%). COVID-19 was the cause of death in 1.745 people of both sexes and contributed 71.3% to excess mortality, the proportion being similar between sexes (71.4% in men and 71.2% in women).

**Figure 3:**
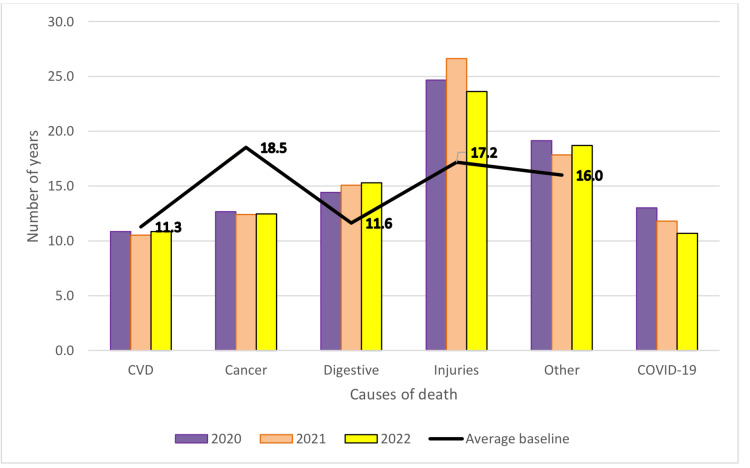
Pandemic period changes in PYLL/death, mean, both sexes PYLL: Potential Years of Life Lost, CVD: Cardiovascular disease, COVID-19: Coronavirus disease 2019

**Figure 4:**
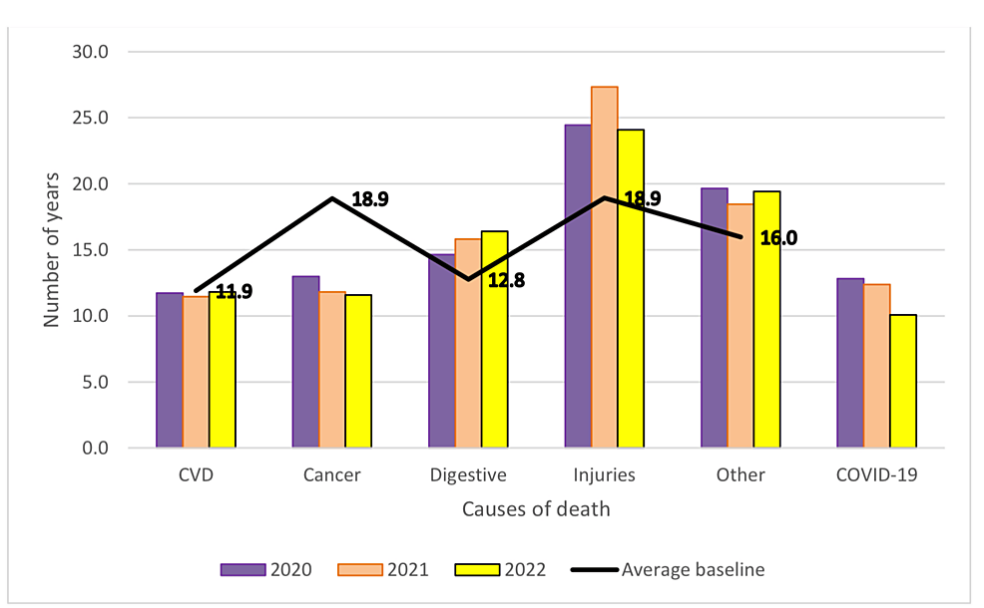
Pandemic period changes in PYLL/death, mean, men PYLL: Potential Years of Life Lost, CVD: Cardiovascular disease, COVID-19: Coronavirus disease 2019

**Figure 5:**
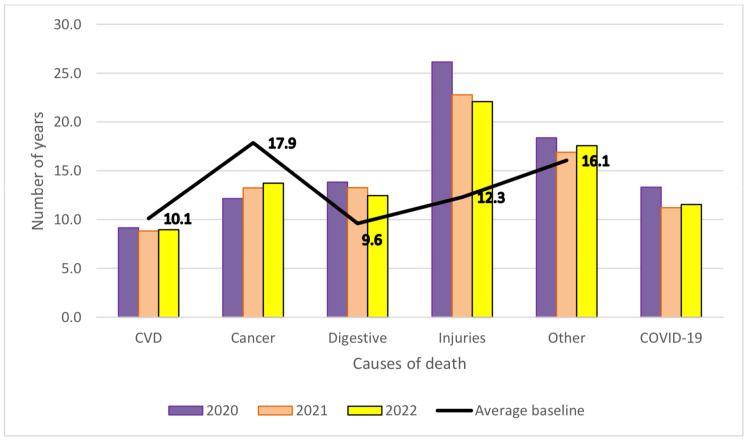
Pandemic period changes in PYLL/death, mean, women PYLL: Potential Years of Life Lost, CVD: Cardiovascular disease, COVID-19: Coronavirus disease 2019

PYLL/causes of death for each year of the pandemic and comparison with the mean baseline are shown in Table 6 and Figure 3 (both sexes), Figure 4 (men) and Figure 5 (women). Compared to the analysed period prior to the pandemic, during the pandemic, there was a decrease of approximately 1 year in PYLL/death by all causes. By group of causes in the case of PYLL/death, there was a decrease during the pandemic compared to the reference period in the case of cardiovascular diseases and cancer, but also an increase in the case of digestive diseases and injuries.

**Table 6:** PYLL/cause of death, total and by genders PYLL: Potential Years of Life Lost, CVD: Cardiovascular disease, COVID-19: Coronavirus disease 2019

PYLL/causes of death	Average baseline	2020	2021	2022
Both sexes				
All deaths	14.6	13.6	13.1	13.6
CVD	11.3	10.9	10.5	10,9
Cancer	18.5	12.7	12.4	12.5
Digestive	11.6	14.4	15.1	15.3
Injuries	17.2	24.7	26.6	23.6
Other	16.0	19.1	17.8	18.7
COVID-19	NA	13.0	11.8	10.7
Deaths excluding COVID-19	14.6	13.6	13.4	13.8
Men				
All deaths	15.1	14.2	13.8	14.1
CVD	11.9	11.7	11.5	11.8
Cancer	189	13.0	11.8	11.6
Digestive	12.8	14.6	15.8	16.4
Injuries	18.9	24.4	27.3	24.1
Other	16.0	19.6	18.5	19.4
COVID-19	NA	12.8	12.4	10.1
Deaths excluding COVID-19	15.1	14.3	14.2	14.3
Women				
All deaths	13.5	12.5	11.8	12.6
CVD	10.1	9.2	8.8	9.0
Cancer	17.9	12.2	13.3	13.7
Digestive	9.6	13.8	13.3	12.5
Injuries	12.3	26.1	22.8	22.1
Other	16.1	18.4	16.9	17.6
COVID-19	NA	13.3	11.2	11.5
Deaths excluding COVID-19	13.5	12.4	12.1	12.7

For COVID-19, PYLL/death showed a downward trend over the duration of the pandemic in both genders (13 in 2020, 11.8 in 2021 and 10.7 in 2022) and in men (12.8 in 2020, 12.4 in 2021 and 10.4 in 2022). In case of women, COVID-19 PYLL/death decreased in 2021 (12.1) compared with 2020 (12.4), but increased again, in 2022 (12.7). Cardiovascular diseases (CVD) ranked as the primary cause of premature death during 2017-2022, regardless of whether we examined the cause of death or PYLL (Table 7). In the pre-pandemic period, malignancies and digestive diseases occupied the second and third positions in terms of the number of deaths, while in terms of PYLL, malignancies remained the second leading cause of death, with injuries ranking in the third place in 2017 and 2018, and digestive diseases in 2019. During the pandemic, COVID-19 was the third leading cause of death in 2020 and 2021; however, in 2022, the last year of the pandemic, the ranking of the top three causes of death looked similar to that of the pre-pandemic years, with COVID-19 not included among the top three causes of death.

**Table 7:** Annual scale of the top three causes of premature death – comparative aspects between the pre-pandemic and pandemic periods PYLL: Potential Years of Life Lost, CVD: Cardiovascular disease, COVID-19: Coronavirus disease 2019

Pre-pandemic period – number of deaths				Pandemic period of COVID-19 – number of deaths			
Rank	2017	2018	2019	Rank	2020	2021	2022
I	CVD	CVD	CVD	I	CVD	CVD	CVD
II	Cancer	Cancer	Cancer	II	Cancer	COVID-19	Cancer
III	Digestive	Digestive	Digestive	III	COVID-19	Cancer	Injuries
Pre-pandemic – PYLL				Pandemic period COVID-19 – PYLL			
Rank	2017	2018	2019	Rank	2020	2021	2022
I	CVD	CVD	CVD	I	CVD	CVD	CVD
II	Cancer	Cancer	Cancer	II	Cancer	COVID-19	Cancer
III	Injuries	Injuries	Digestive	III	COVID-19	Cancer	Injuries

## Discussion

The impact of COVID-19 on overall mortality is well-studied. The highest number of deaths due to COVID-19 was reported in 2021 [1]. The use of PYLL to assess the impact of the COVID-19 pandemic on the population is described in the literature in numerous studies [5,8,11,17-19]. In a time interval situated between the beginning of the pandemic and the end of March, in Bulgaria the burden of the COVID-19 pandemic was quantified as “12.57, 12.02 and 12.51 years of life lost overall, for males, and for females, respectively, based on the PYLL metric” [8]. In an international comparison made for the period between the beginning of the pandemic and August 2020, “the average PYLL per death was 8.7 years, with substantial variation ranging from 2.7 years in Australia to 19.3 PYLL in Ukraine” [5]. For the whole year 2020, they have been identified as “countries with an average PYLL ≥13 are Estonia, Lithuania, Serbia, and Bulgaria, compared to values in the 7 to 9.5 years range for countries such as Switzerland, Sweden, and Belgium” [17]. An Australian study comparing the 2009 H1N1 flu epidemic with the 2020 winter COVID-19 pandemic, shows that, although the crude death rate in-hospital was similar and due to the demographic differences of the affected patients, in-hospital deaths due to H1N1 influenza caused 11 times more PYLLs compared to COVID-19, in critically ill patients [18]. Starting from the calculation of PYLL using age limits of 70, 75, and 80 years and comparisons between Italy, Germany, and the USA of the PYLL indicators for COVID-19, a study carried out in 2000 recommends setting the age limit at 80 years for calculating the impact of COVID-19 at the population level [19]. The study carried out in Hungary, regarding a period from the beginning of the pandemic until May 12, 2021, “showed 10.5 years of life lost for each death” [11] for COVID-19 cases.

There are many studies quantifying excess mortality and PYLL by cause of death in the pandemic years of 2020 and 2021 [20-22]. Globally, more than 20.500.000 years of life were lost because of COVID-19, this information being provided by a study developed on 81 countries in a time interval between 2015 and 2020 [23]. Using data from the Swedish National Patient Register, one study [20] estimated the impact of the COVID-19 pandemic on age and gender-specific excess mortality and PYLL in Sweden for the year 2020 and the first five months of 2021 by comparison with relevant mortality data recorded between 2017-2019. Another study, referring to the year 2020, estimates weekly excess of all-cause mortality in Norway and Sweden, Years of Life Lost (YLL) assigned to COVID-19 in Sweden, and the significance of the change in mortality [21]. By measuring years of life lost (YLL) imputable to the pandemic, directly or indirectly, comparing mortality across geographic and socioeconomic groups, another study performed in England and Wales [12] showed that in the first COVID-19 pandemic year, strong socioeconomic and geographic inequalities came to become a reality and the most deprived areas reported the highest number of PYLL. To the best of our knowledge, no studies have been performed regarding the impact of COVID-19 on premature mortality and excess PYLL for the entire pandemic period (2020-2022). Data provided by our study regarding the entire pandemic period showed that only in Bihor County, Romania, 20,921 years of lives were lost because of COVID-19 disease.

According to a recent study (Williams G. et al, 2022) [24], Romania did not furnish information regarding sex distribution during the pandemic. The study we conducted provided information about this aspect, so in Bihor County, Romania, men were more affected compared to women by COVID-19, and PYLL in cases of males was higher compared with females. Despite the geographical limitation of our study to Bihor County, Romania, it provides high-quality data for future investigations. This is significant since, to the best of our knowledge, there exists no other demographic dataset that can provide substantial source of information on PYLL across both pre-pandemic and pandemic periods.

Limitations of the study

We acknowledge the limitations of using PYLL (setting the norm for which PYLL is equal to zero, recording deaths from a specific cause, interpreting results, focusing on deaths, and ignoring the quality of life), but most authors recommend using this indicator. Another limitation of this study could be the rectitude of cause of death coding.

## Conclusions

The data obtained demonstrate that in the studied population (deaths from Bihor County, Romania) there was an excess of premature mortality during the pandemic (2020-2022) compared to the pre-pandemic period (2017-2019) and in the case of men the excess was higher in both number of deaths and PYLL compared to women. Much of the excess of premature mortality is attributed to COVID-19, even if throughout the analysed period, cardiovascular diseases represented the main cause of death. In the last year of the pandemic (2022) the hierarchy of causes of premature mortality was similar to the pre-pandemic years. When analysing PYLL/death by group causes, a decrease in cardiovascular diseases and malignancies was observed during the COVID-19 pandemic compared with a pre-pandemic period, but conversely, an increase was noted in digestive diseases and injuries. The excess of premature deaths in the pandemic period and the decrease in PYLL/death express a higher proportion of deaths at ages close to the defined cut-off of 75 years. Additional investigations will be conducted into PYLL and excess of premature mortality regarding the COVID-19 pandemic nationwide in Romania.
